# An image classification approach to analyze the suppression of plant immunity by the human pathogen *Salmonella* Typhimurium

**DOI:** 10.1186/1471-2105-13-171

**Published:** 2012-07-19

**Authors:** Marek Schikora, Balram Neupane, Satish Madhogaria, Wolfgang Koch, Daniel Cremers, Heribert Hirt, Karl-Heinz Kogel, Adam Schikora

**Affiliations:** 1 Department Sensor Data and Information Fusion, Fraunhofer FKIE, 53343 Wachtberg, Germany; 2 Institute for Plant Pathology and Applied Zoology, IFZ, JL University Giessen, 35392 Giessen, Germany; 3 Computer Science Department, Technical University of Munich, 85748 Garching, Germany; 4 URGV Plant Genomics, INRA/CNRS/University d’Evry, 97000 Evry, France

## Abstract

**Background:**

The enteric pathogen *Salmonella* is the causative agent of the majority of food-borne bacterial poisonings. Resent research revealed that colonization of plants by *Salmonella* is an active infection process. *Salmonella* changes the metabolism and adjust the plant host by suppressing the defense mechanisms. In this report we developed an automatic algorithm to quantify the symptoms caused by *Salmonella* infection on *Arabidopsis*.

**Results:**

The algorithm is designed to attribute image pixels into one of the two classes: healthy and unhealthy. The task is solved in three steps. First, we perform segmentation to divide the image into foreground and background. In the second step, a support vector machine (SVM) is applied to predict the class of each pixel belonging to the foreground. And finally, we do refinement by a neighborhood-check in order to omit all falsely classified pixels from the second step. The developed algorithm was tested on infection with the non-pathogenic *E. coli* and the plant pathogen *Pseudomonas syringae* and used to study the interaction between plants and *Salmonella* wild type and T3SS mutants. We proved that T3SS mutants of *Salmonella* are unable to suppress the plant defenses. Results obtained through the automatic analyses were further verified on biochemical and transcriptome levels.

**Conclusion:**

This report presents an automatic pixel-based classification method for detecting “unhealthy” regions in leaf images. The proposed method was compared to existing method and showed a higher accuracy. We used this algorithm to study the impact of the human pathogenic bacterium *Salmonella* Typhimurium on plants immune system. The comparison between wild type bacteria and T3SS mutants showed similarity in the infection process in animals and in plants. Plant epidemiology is only one possible application of the proposed algorithm, it can be easily extended to other detection tasks, which also rely on color information, or even extended to other features.

## Background

Numerous bacteria, pathogenic to humans and other mammals, are found to thrive also on plants, *Salmonella enterica, Pseudomonas aeruginosa, Burkholderia cepacia, Erwinia spp., Staphylococcus aureus, Escherichia coli* O157:H7, and *Listeria monocytogenes* are able to infect both animal and plant organisms [1-5]. Among these, *Salmonella*, a genus of Gram-negative enteropathogenic bacteria, are the causal agents of both gastroenteritis and typhoid fever. They are responsible for an estimated one million casualties and about 100 million human infections annually. Not only in developing countries in Africa or South-East Asia, where typhoid and paratyphoid fever are unfortunately still prevalent, but also in developed communities salmonellosis is still not vanquished. The most common mode of infection in humans is by ingestion of contaminated food or water.

### Plants can be the source of infection

Many reports have linked food poisoning with the consumption of *Salmonella*-contaminated raw vegetables and fruits (for review see [2,6]). A large study conducted in the European Union revealed that in 2007, 0.3% of products were infected with *Salmonella* bacteria [7], during the same time in UK, the Netherlands, Germany, and Ireland 0.1 to 2.3% of pre-cut products were contaminated [7]. In the USA, the proportion of raw food-associated salmonellosis outbreaks increased from 0.7% in the 1960s to 6% in the 1990s [8], and crossed 25% in recent years [9]. In order to monitor the molecular subtype pattern of the outbreak strains a national program (PulseNet) was created in the USA [10]. This program significantly improved the identification of outbreaks and their sources. Most studies on *Salmonella*-plant interactions suggested an epiphytic lifestyle of *Salmonella* on plants. However, a growing body of evidence points to an active process in which bacteria infect various plants and use them as viable hosts [11-20]. In this report we developed an automatic algorithm to quantify the symptoms caused by *Salmonella* infection on *Arabidopsis* plants. The algorithm is designed to attribute image pixels into one of the two classes: healthy and unhealthy. We show that it outperforms other algorithms developed for this task. It was tested on infection with the non-pathogenic *E. coli* and the plant pathogen *Pseudomonas syringae* and subsequently used to study the interaction between plant host and *Salmonella* wild type and T3SS mutants. We proved that T3SS mutants of *Salmonella* are unable to suppress the plant defense mechanisms. Results obtained through the automatic analyses were further verified on biochemical and transcriptome levels.

### Automatic classification as key concept to objective analysis

During the last few years, image classification has proved increasingly useful in biology, as numerous tasks have been simplified with the help of automated image classification [21-23]. Plant diseases need to be controlled for at least two reasons: to maintain the quality of food produced by farmers around the world and in order to reduce the food-borne illnesses originated from infected plants [24]. Thus, automatic identification of “unhealthy” regions in leaf images is a useful tool for various biological research projects aiming the control of diseases or characterization of plant defense mechanisms [25,26]. There is a wide variety of plant diseases caused by either environmental factors (nutrition, moisture, temperature, *etc.*) or by other organisms (fungi, bacteria, viruses). However, in most cases the common symptom is the change of the leaf color. A good color variation model can be employed to distinguish “healthy” and “unhealthy” regions in leaf images. A probabilistic algorithm, employing a Gaussian mixture model (GMM) and a Bayesian classifier to classify disease symptoms in *Arabidopsis* plants was presented in [27]. However, because the estimation of a robust GMM is not always possible from the real data, results from Bayes-like classifiers can be inaccurate. To overcome this limitation we propose a different classification strategy. The algorithm described in this report uses color feature space as input for learning algorithm (Support Vector Machine (SVM)) which classifies the pixels of leaf images.

## Biological Background

### Type III secretion system is responsible for effectors delivery

Salmonellosis develops after the bacteria enter epithelial cells of the intestine [28]. Studies of the infection mechanisms in animals have shown that *Salmonella* actively remodel the host cells physiology and architecture, and suppress the host immune system by injecting a cocktail of effectors delivered by Type III Secretion Systems (T3SSs). *Salmonellaenterica* subsp. *enterica* has two distinct T3SSs, T3SS-1 and T3SS-2, encoded by the *Salmonella* Pathogenicity Islands (SPI) SPI-1 and SPI-2, respectively [29,30]. T3SS-1 secretes at least 16 proteins of which 6 were shown to interact with the host signaling cascades and the cytoskeleton. T3SS-2 secretes at least 19 *Salmonellaenterica*-specific effector proteins that are involved in survival and multiplication within the host cell [31,32]. The expression and the secretion of SPI-1 and SPI-2 encoded effectors are tightly regulated. Recently, a sorting platform for T3SS effectors was reported that determines the appropriate hierarchy for protein secretion [33]. In this study, the authors identified the cytoplasmic SpaO-OrgA-OrgB complex, which enables the sequential delivery of translocases before the secretion of the actual effectors. Furthermore, the authors described the role of specific chaperones in the recognition and loading of effectors into the sorting SpaO-OrgA-OrgB complex. In conclusion, it was postulated that similar sorting platforms might exist in other T3SSs as their components are widely conserved. Many recent reports suggest that the mechanisms used by *Salmonella* to infect animal and plant hosts might be similar [20,34].

### Effector proteins defeat immune system

In the battle between pathogen and its host, the pathogen needs to suppress the host immune system in order to establish a successful infection. The early line of immunity relies on the recognition of conserved pathogen-associated molecular patterns (PAMPs) by host-encoded pattern recognition receptors (PRRs) and thereby the activation of an array of defense responses called PAMP-triggered immunity (PTI). The best-studied PAMP in plants is flg22, a conserved 22 amino acid peptide from the bacterial flagellar protein flagellin, recognized by the PRR FLAGELLIN INSENSITIVE 2 (FLS2) [35]. During infection, pathogens secrete effectors with the aim to suppress PTI and cause effector-triggered susceptibility (ETS). In a second layer of defense, intracellular resistance proteins (R-proteins) recognize pathogen effectors and activate effector-triggered immunity (ETI). The plant pathogen *Pseudomonas syringae* injects about 40 effectors into plant cells. Among these, AvrPto, AvrPtoB and HopAI1 attenuate the flg22-induced defense responses [36-38]. Strikingly, HopAI1 is also present in animal/human pathogens such as *Shigella spp.* (OspF) [39,40] and *Salmonella spp.* (SpvC) [41], where it interacts with the mitogen-activated protein kinases (MAPKs) ERK1/2 and p38. The role of multiple *Salmonella* effectors in animal infection has been described (reviewed in [42]), but a functional proof of *Salmonella* effector action in plants is still missing. Nonetheless, several lines of evidence point to an active interaction between these bacteria and plant hosts.

### *Salmonella* suppresses plant defenses

Two very recent studies report the suppression of the plant immune system by *Salmonella*[34,43]. The authors showed that in contrast to wild type living bacteria, dead or chloramphenicol treated bacteria elicited an oxidative burst and pH changes in tobacco cells. A similar response was provoked by the *invA*^−^ mutant, which has no functional SPI-1 T3SS [34]. Those results suggest that *Salmonella* depends on the secretion of effectors during plant infection and actively suppresses the immune response. We observed similar phenomena during infection of *Arabidopsis*[43]. *Salmonella* T3SS mutants were compromised in virulence towards the wild type *Col*-0 plants. Comparison between global transcriptome profiles of *Arabidopsis* plants infected with wild type *Salmonella* or the *prgH*^−^(T3SS-1) mutant revealed 649 genes, which are upregulated upon challenge with *prgH*^−^ mutant but not with the wild type *Salmonella*. GO term enrichment analysis (AmiGO version 1,7) [44] of these 649 *prgH*^−^-specific genes showed an overrepresentation of genes related to responses to biotic stress, relations with other organisms and defense mechanisms [43]. Moreover, challenge with T3SS mutants provoked stronger symptoms on *Arabidopsis* plants suggesting that those mutants are not able to suppress plant defenses. Those symptoms could be, at least to some extent, part of the hypersensitivity response (HR). HR is a common defense mechanism against biotrophic and hemibiotrophic pathogens, resulting in localized cell death and therefore arresting the proliferation of pathogen. However, successful pathogenic bacteria evolved mechanisms to suppress this resistance mechanism. In a simplified manner one could describe a very fast and strong occurrence of chlorotic and dead tissues after infection with *Salmonella* as resistance mechanism. On the other hand, necrotic and lysed tissues suggest no resistance capabilities. This distinction served as the base for an automatic analysis of infection symptoms caused by wild type *Salmonella* and four distinct T3SS mutants as well as the plant pathogenic *Pseudomonas syringae* and the nonpathogenic *E. coli*.

## Image-Based Classification

**Figure 1:**
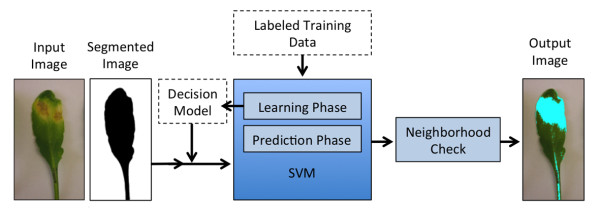
**Overview of the proposed algorithm.** Input image is a *Arabidopsis* leaf with almost monochromatic background. First, segmentation method is applied to obtain the pixels belonging to the leaf. Second, each pixel belonging to the leaf is classified using linear SVM classifier. Finally, the output from classifier is further refined through neighborhood-check method to obtain the output image.

A good color variation model can be employed to distinguish “healthy” and “unhealthy” regions in leaf images. A probabilistic algorithm, employing a Gaussian mixture model (GMM) and a Bayesian classifier for classifying disease symptoms in *Arabidopsis* plants was presented in [27]. However, results from Bayes-like classifiers can be inaccurate, because the estimation of a robust GMM is not always possible from real data. To overcome these limitations we propose here a different classification strategy. The algorithm described in this paper uses color feature space as input to a well-known machine learning algorithm (Support Vector Machine (SVM)) to classify the pixels of a leaf image. Figure 1 presents an overview of the steps described in this paper. First a segmentation method, described in section Segmentation, is applied to obtain a binary image with only foreground and background information. Each pixel belonging to the foreground region is then given as an input to a linear SVM classifier (described below) to predict the class to which it belongs. After identification of all pixels belonging to the foreground, the neighborhood information is used to alter the result of pixels classified as “unhealthy”. The following neighborhood-check method is described in section Neighborhood. Parts of this work have been previously published in [45].

### Segmentation

In the first step, we needed to separate the pixels belonging to a leaf (foreground) and not belonging to the leaf (background) in the input image. The input used in this study were leaf images with almost monochromatic background. Besides reducing the computational cost in the next step, a good segmentation method can also improve the overall result by eliminating any misclassification outside the leaf boundary. Therefore, we divide the image into foreground and background so that only the pixels belonging to the foreground are considered for classification in the next step. The binary segmentation of an image $I\text{:}\Omega \to {\left[0,1\right]}^{3}\subset {\mathbb{R}}_{1}^{3}$ with $\Omega \subseteq {\mathbb{R}}_{1}^{2}$ can be seen as separation of the image plane *Ω*into disjoint regions *Ω*_obj_and *Ω*_bgd_, with *Ω* = *Ω*_obj_∪ *Ω*_bgd_ ∪*Γ*, where *Γ*denotes the contour of the segmentation. So we are looking for a binary image *u*:*Ω* → {0,1}. The most influential region based image segmentation model was introduced by Mumford and Shah in 1989 [46]. Many models based on this functional and its derivatives have been proposed, e.g. [47,48]. In this study, we used the segmentation method proposed in [27]. The method uses a convex energy functional [49] but with the I1I2I3 color space [50] instead of HSV. Following [49] a convex energy functional in the I1I2I3 color space can be written as:

(1) $$\begin{array}{lll} \;E(u,\mu_{\text{obj}},\mu_{\text{bgd}}) & =\underset{\Omega }{\int }\left(f(I_{123}\left(x\right),\mu_{\text{obj}})-f(I_{123}\left(x\right),\mu_{\text{bgd}})\right)\; & \;\\ & \;\times u(x)dx+\lambda \underset{\Omega }{\int }|\nabla u(x)|dx,\; \end{array}$$

with

(2) $$\begin{array}{lll} f(I_{123}(x),\mu ) & =w_{1}{\left({\left[I_{123}(x)\right]}_{\text{I}1}-\mu_{\text{I}1}\right)}^{2}\; & \;\\ & \;+w_{2}{\left({\left[I_{123}(x)\right]}_{\text{I}2}-\mu_{\text{I}2}\right)}^{2}\; & \;\\ & \;+w_{3}{\left({\left[I_{123}(x)\right]}_{\text{I}3}-\mu_{\text{I}3}\right)}^{2}\; \end{array}$$

denoting a weighted squared sum of the individual channels. For the results presented in this paper we used *w*_I1_ = 0.1 and *w*_I2_ = *w*_I3_ = 0.45. As an additional input we used mean values for the foreground ***μ***_obj_and background ***μ***_bgd_ and a smoothing parameter λ∈ℝ. [*I*_123_(**x**)]_I*n*_ denotes the value of pixel **x** for the color channel *I*_*n*_. The desired segmentation is a binary image $u\text{:}\Omega \subseteq {\mathbb{R}}^{2}\to \{0,1\}$. We minimize (1) for real-valued *u* using successive over-relaxation (SOR), as in [49,51] and binarize the solution to obtain the globally optimal segmentation.

### SVM classification

Having obtained a binary image $u\text{:}\Omega \subseteq {\mathbb{R}}^{2}\to \{0,1\}$, we classified each pixel belonging to *Ω*_obj_ into “unhealthy” or “healthy” regions. For this purpose we use a state-of-the-art machine-learning algorithm, support vector machine (SVM), that have found a wide acceptance in recent years due to its ability to classify linear and non-linear data. SVMs have been applied with great success in many challenging classification problems processing large data sets. The basic concept was introduced in [52]. In our work we will use a modified maximum margin idea, called Soft Margin, which allows the handling of not perfectly linear separable data. It is based on learning from examples, which means, it requires a separate set of training and testing data. The training algorithm builds a model that predicts the class of unknown input data.

We needed a labeled training data, which serves as an input for the learning function. For training we chose 40.000 pixels of leaf images randomly from all available images. Then we hand-labeled every chosen pixel into one of three classes: healthy, unhealthy and background. Like many other pixel-based classification methods, we exploit the color variation property of image co-ordinates in order to form a decision model. Since the components of I1I2I3 color space [50] are uncorrelated, statistically it is the best way to detect color variations. While I1 contains the illumination information, I2 and I3 mainly contain color information. Hence, we used only I2 and I3 in order to provide invariance to illumination changes. Thus the training data comprise of 2D color values, selected from “healthy” and “unhealthy” leaf images and labeled into the two different classes.

#### Training phase - offline

**Figure 2:**
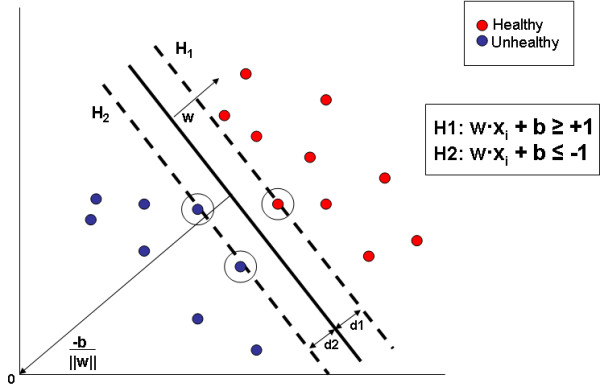
**Hyperplane.** Hyperplane through two linearly separable classes. Points on the hyperplanes are called support vectors and form the basis for predicting the class of unlabeled data.

Suppose we have *L* number of training vectors belonging to two different classes, (**x**_*i*_,*y*_*i*_) where *i* = 1,…,*L* and *y*_*i*_is either 1 “healthy” or -1 “unhealthy”, indicating the class to which **x**_*i*_ belongs. SVM is based on the concept of finding a hyperplane which can be described by a set of points satisfying the equation:

(3) $$w\cdot x+b=0,\;\;\;w\in {\mathbb{R}}^{n},\;x\in {\mathbb{R}}^{n},\;b\in \mathbb{R}$$

where **w** is normal to the hyperplane and *b*/||**w**|| is the perpendicular distance from the hyperplane to the origin. The goal here is to choose **w** and *b* so as to maximize the margin between two parallel hyperplanes H1 and H2 (see Figure 2). Thus, our training data can be described by equation:

(4) $$y_{i}(w\cdot x_{i}+b)-1\geq 0\;\forall_{i}$$

Considering the Soft Margin idea we can reformulate (4) as

(5) $$y_{i}(w\cdot x_{i}+b)-1+\xi_{i}\geq 0\;\forall_{i},$$

with slack variables *ξ*_*i*_, which measure the degree of misclassification of the data **x**_*i*_.

The training part (Additional file 1: Figure S1) of SVM algorithm finds a **w**that leads to the largest *b*. It can be solved by finding the solution of following optimization problem:

(6) $$\;\underset{w,\xi ,b}{\min}\;\left\{\;\frac{1}{2}\left|\right|w{\left|\right|}^{2}\;+\;C\underset{i}{\sum }\xi_{i}\;\right\}\;\text{such that}\;y_{i}(w\cdot x_{i}+b)-1+\xi_{i}\;\geq \;0\forall_{i}$$

It is transformed into its dual form by using Lagrangian formalization:

(7) $$\;\begin{array}{ll} L(w,b,\alpha ,\xi ,\beta ) & =\frac{1}{2}||w|{|}^{2}\;+\;C\underset{i}{\sum }\xi_{i}\;-\;\overset{L}{\underset{i=1}{\sum }}\alpha_{i}\left[y_{i}(w\cdot x_{i}+b)\right)\\ & \;-\left(1+\xi_{i}\right]-\underset{i}{\sum }\beta_{i}\xi_{i} \end{array}$$

where *α*_*i*_, *β*_*i*_ are non-negative Lagrange multipliers. According to [53], the final dual optimization problem can be written as:

(8) $$\begin{array}{c} \text{maximize}\;\;L_{D}=\overset{L}{\underset{i=1}{\sum }}\alpha_{i}-\frac{1}{2}\underset{\mathrm{ij}}{\sum }\alpha_{i}\alpha_{j}y_{i}y_{j}x_{i}^{T}x_{i}\\ \text{subject to}\underset{i}{\sum }\alpha_{i}y_{i}=0\;\;\text{and}\;\;0\leq \alpha_{i}\leq C\;\;\forall_{i} \end{array}$$

Note that the dual form requires only the dot product of each input vector **x**_*i*_ to be calculated. Equation (8) is a convex optimization problem and QP (Quadratic Programming) solver is run on it in order to obtain *α*, from which we can get **w**:

(9) $$w=\overset{L}{\underset{i=1}{\sum }}\alpha_{i}y_{i}x_{i}$$

The training cases with *α*_*i*_ > 0 are called support vectors, or sometimes margin points, they determine the solution. Any data point which is a support vector will have the following form:

(10) $$y_{s}(w\cdot x_{s}+b)=1$$

Using any support vector, *b* can be derived from equations 9 and 10 (see [53,54] for detailed derivation):

(11) $$b=\underset{s\in S}{\sum }(y_{s}-\underset{m\in S}{\sum }\alpha_{m}y_{m}x_{m}.x_{s})$$

Where *S* denotes the set of indices of the support vectors. *S* is determined by finding the indices *i* where *α*_*i*_ > 0. Instead of using an arbitrary support vector **x**_*s*_, it is better to take an average of the support vectors in *S*. Thus, the training phase of SVM gives **w** and **b** which is used later to compute the class of unknown vectors. Since the training phase is time consuming, it is done offline.

#### Prediction phase - online

In the prediction phase, all pixels labeled as foreground pixel in the segmentation step are classified into one of the two classes - “healthy” or “unhealthy”. Each new pixel, **x**^*′*^ is classified by evaluating:

(12) $$y^{\prime }=\text{sign}(w\cdot x^{\prime }+b)$$

where **w** and *b* are obtained from the training part of the SVM algorithm.

**Figure 3:**
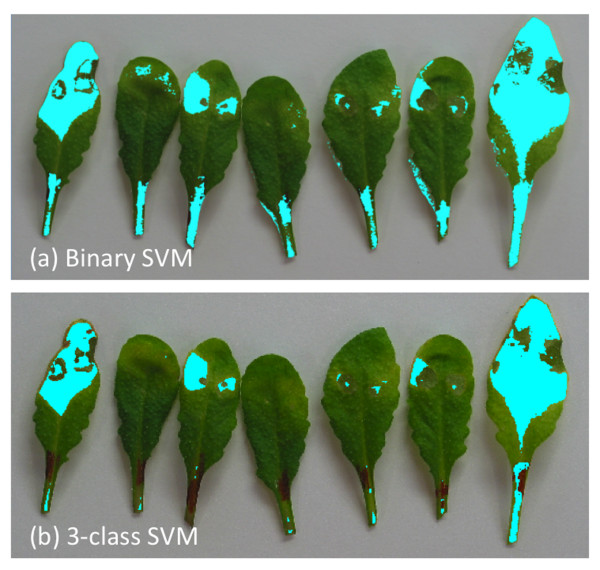
**Multi-class SVM.** Top image **(a)** shows output from the binary SVM classifier, where unhealthy pixels outside the leaf boundary are noticeable. This is due to prominent shadow near the leaf boundary which is labeled as foreground pixels in the segmentation step. We can overcome this problem by using a multi-class SVM **(b)**, where each pixel is classified into three classes: healthy, unhealthy and background.

Although, using binary SVM gives good performance in most of the cases, it still relies on a good segmentation method in step 2, which means that if pixels are labeled as foreground outside the boundary of the leaf then the SVM should also classify them into one of the two classes. As an example in Figure 3, we can see that due to an error in the segmentation, there are pixels outside the leaf region marked as “unhealthy”. Segmentation error occurs when a prominent shadow of the leaf is present in the image, due to which the proposed segmentation method labels pixels inside the shadow region as foreground. To make the SVM classifier more efficient we can classify each pixel into one of the three classes: “healthy”, “unhealthy” and background. Inherently, SVMs are binary classifiers it is however easily possible to do a multi-class classification with SVMs by building a set of one-verses-one classifiers. In this approach, classification is done by a max-wins voting strategy, in which every classifier assigns the instance to one of the two classes, then the vote for the assigned class is increased by one vote, and finally the class with the most votes determines the instance classification. Figure 3 compares the result with two-class and three-class SVM.

### Neighborhood-Check

Output from the classification step shows a high number of isolated pixels labeled as “unhealthy”, which maybe be perceived by human eye as without any symptoms. This is due to the fact that single pixel is too small for an human eye to be recognized and usually we see a combination of pixels. Another possibility could be a pixel within an “healthy” region that have similar color values as the one from infected region which makes the classifier to mark it as “unhealthy” one. Here, we exploit the fact that usually the infected regions are densely populated with infected pixels. We can, therefore, use the neighborhood classification information to alter the result of isolated pixels, classified as “unhealthy”. This step works as follows: For each (**x**_*i*_,*y*_*i*_) with *y*_*i*_ = −1 (unhealthy), define the number of pixels which are classified as unhealthy in the neighborhood radius n∈ℤ as *c*_*i*_. We perform the following:

(13) $$\mathrm{if}\;c_{i}<\frac{{\left(2n+1\right)}^{2}-1}{2},\;\text{then set}\;y_{i}=+1\;\text{(healthy)}$$

**Figure 4:**
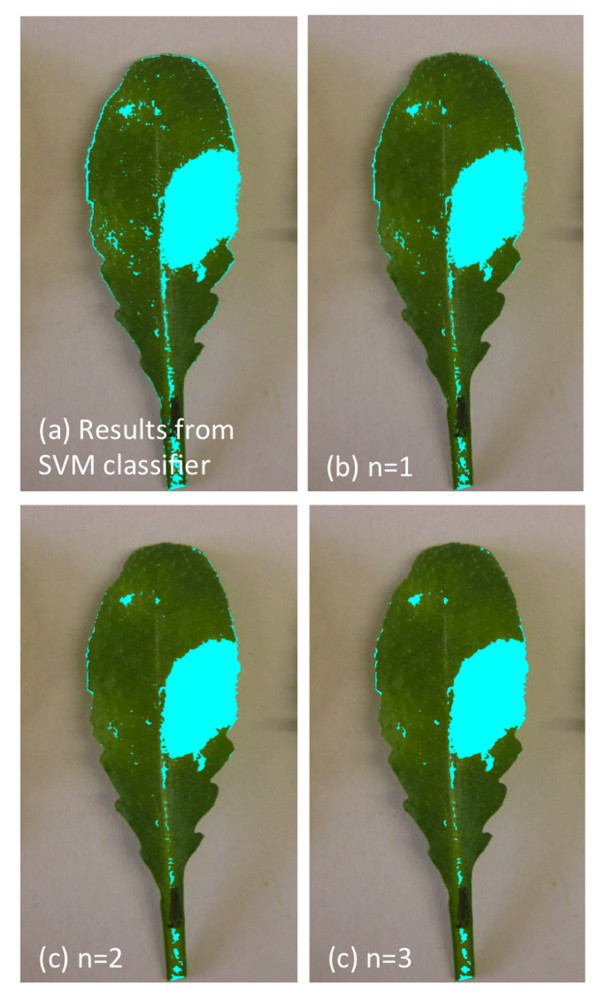
**Different radius parameter used in this study.** Neighborhood radius could be varied to obtain better result. We can see from the figure that neighborhood radius, n = 2 and n = 3 yields almost the same result. Using n = 1 improves the result from SVM classifier, **(a)** but not as good as **(b)** and **(c)**.

**Figure 5:**
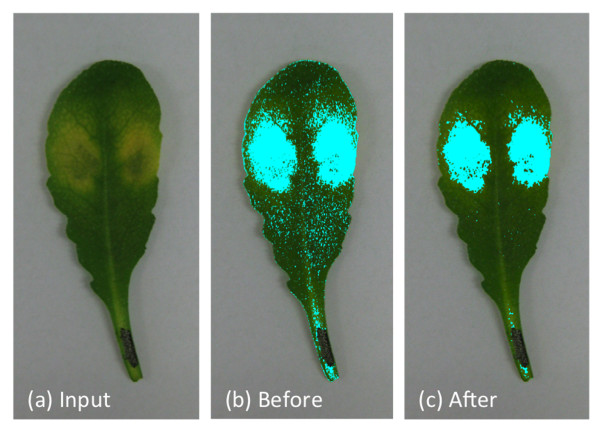
**Neighborhood check.** Input image is shown in **(a)**, **(b)** is the output from SVM classifier. It shows high number of pixels marked as unhealthy while the human eye perceive them as healthy. In an attempt to alter the result of those isolated pixels, neighborhood-check method is applied. **(c)** is the result from neighborhood-check and matches well with the visual perception of human observer.

We used *n* = 2 to obtain the results presented in this report, because using neighborhood radius of n = 1 slightly improves the result from SVM classifier though not as good as using 2 or 3. Although neighborhood radius of 2 or 3 shows almost the same effect, we choose n = 2 to reduce the computational cost. Figure 4 shows the effect of using n = 1, 2 and 3. Figure 5 shows another example where the result from step 2 could be improved remarkably with the help of the neighborhood-check.

### Classification Results

**Figure 6:**
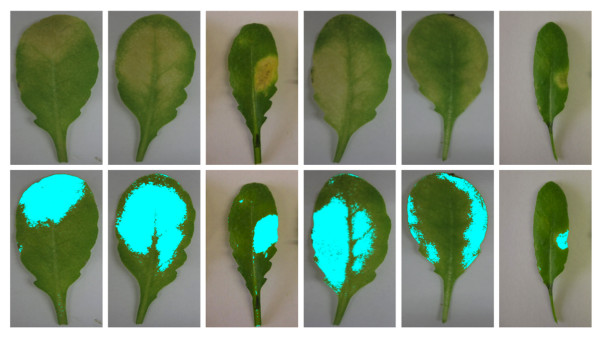
**Classification Results.** Top row shows input images and the bottom row shows outputs from the proposed classification algorithm.

**Figure 7:**
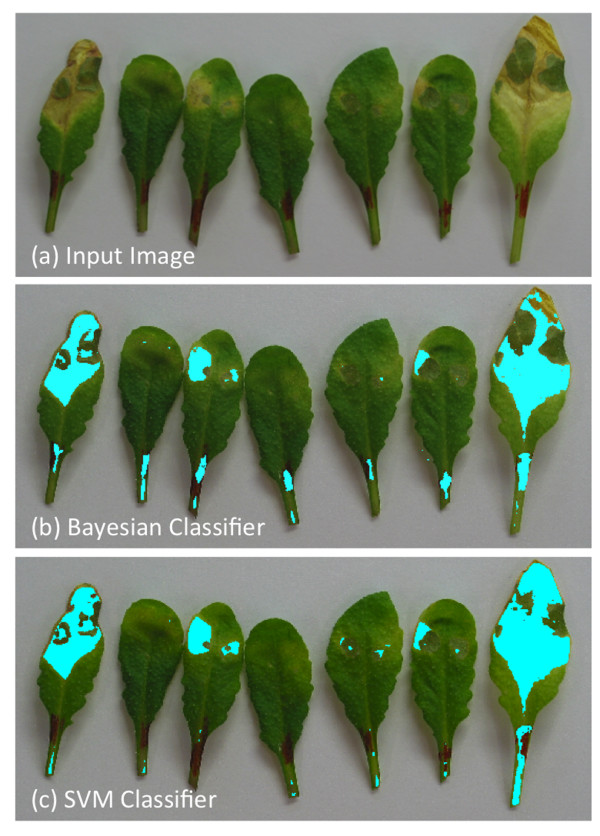
**Comparison between proposed and probabilistic approach.** An example image showing result from probabilistic [27] and the proposed SVM classification. Difference is clearly noticeable in the right-most leaf in the image, where leaf portions are left unmarked by Bayesian classifier. Also pixels outside the leaf boundary (see second from right in **(b)**) are marked. Higher accuracy can be achieved by using the SVM classifier **(c)**.

The classification algorithm has been tested extensively on more than 1200 images of infected leaves. The input images were images of infected leaves with nearly monochromatic background and the output is the classified image with marked “unhealthy” regions. It also provides an objective measurement for the disease rate. Figure 6 shows some outputs from the classification algorithm described above. The results obtained from this algorithm were convincing and could be easily used for biological experiments. Figure 7 shows a comparison between the proposed and a probabilistic method [27]. We extended the probabilistic algorithm with the proposed neighborhood-check to have a fair comparison. The proposed algorithm, which combines the accuracy of SVM with a neighborhood-check method, outperforms the probabilistic method. The Bayesian classifier leave some unhealthy region in leaf unmarked. Moreover, there are some marks near the boundary of the leaf which are wrongly classified as unhealthy. These problems are overcome by using multi-class SVM. SVMs are more robust in separating those data. Experiments prove that higher accuracy could be achieved with SVM. Here, we use linear SVM because it is computationally efficient and avoids the complexities of tuning several parameters, which is the case of non-linear kernels.

In addition, we split 9797 data points from the labeled training set and classified this data to get an objective performance measure. The GMM approach reached a correct classification rate of 91.5%. The proposed SVM approach could improve the results, so that a correct classification rate of 95.8% could be achieved.

## Results

### Photo-based analysis of symptoms caused by different bacteria in *Arabidopsis*

**Figure 8:**
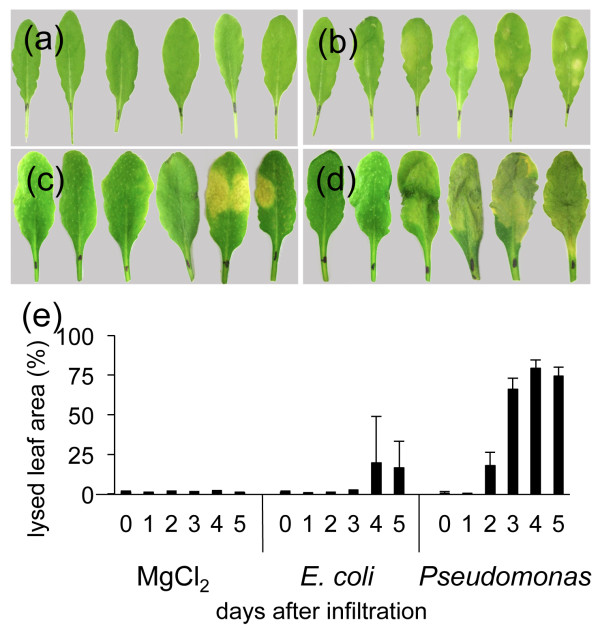
**Symptoms caused by different bacteria.** Analysis of symptoms caused by the non-pathogenic *E. coil* K12 strain DH5*α*and the pathogenic *Pseudomonas syringae* pathovar *tomato* DC3000. Leaves from 6-week-old Arabidopsis plants were infiltrated with bacterial solution at OD_600_ = 0.1. a-d: Macroscopic observations of symptom development from 0 to 5 day after infiltration (DAI) with **(a)**: water (mock control), **(b)**: 10 mM MgCl_2_ (buffer control), **(c)**: *E. coli*, **(d)**: *Pseudomonas syringae*. **(e)**: Calculated average percentages of leaf surfaces showing infection symptoms. Five leaves per time point were photographed. Experiment was repeated five times.

In order to test the algorithm described above, we performed first infections with two bacteria of known virulence towards *Arabidopsis*. We used the nonpathogenic *E. coli* K12 DH5*α*strain and the virulent *Pseudomonas syringae* pathovar *tomato* DC3000 strain as controls. Bacteria were cultivated until early logarithmic phase, washed in 10 mM MgCl_2_, the infiltration solution was adjusted to OD_600_ = 0.1 and syringe-infiltrated into *Arabidopsis* leaves. *Arabidopsis* plants were observed during 5 days after infiltration (DAI), detached leaves were photographed and without any further processing sent to the computing algorithm. As expected the control infiltration with 10 mM MgCl_2_ provoked only slight symptoms in *Arabidopsis* leaves (Figure 8b). Similarly, infiltration with *E. coli* provokes visible symptoms only after 4 DAI (Figure 8c). On the contrary, the virulent *Pseudomonas* strain causes visible necrotic lesions already at 2 DAI, at 4 DAI symptoms reach almost the totality of leaf surfaces (Figure 8d). Calculations made on the base of photos, reflect perfectly the macroscopic observations (Figure 8e).

### T3SS mutants cause stronger symptoms than the wild type bacteria

**Figure 9:**
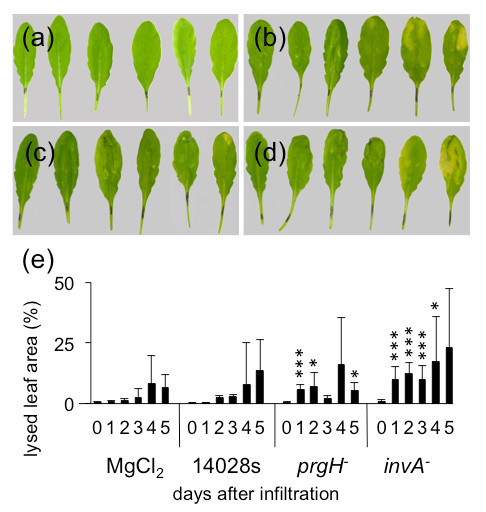
**Symptoms caused by T3SS-1 mutants.** T3SS-1 mutants cause more pronounced symptoms in *Arabidopsis* leaves. Wild type *Salmonella* or mutants in the SPI-1 encoded T3SS were infiltrated into *Arabidopsis* leaves; symptoms were analyzed during 5 DAI. a-d: Macroscopic observations of symptoms development from 0 to 5 DAI with **(a)**: 10 mM MgCl_2_ (buffer control), **(b)**: wild type 14028s strain, **(c)**: *prgH*^−^mutant, **(d)**: *invA*^−^mutant. **(e)**: Calculated average percentages of leaf surfaces showing infection symptoms. Five leaves per time point were photographed. Experiment was repeated five times. ∗*p* ≤ 0.05;∗∗*p* ≤ 0.005;∗∗∗*p* ≤ 0.0005 (Student’s *t* test).

**Figure 10:**
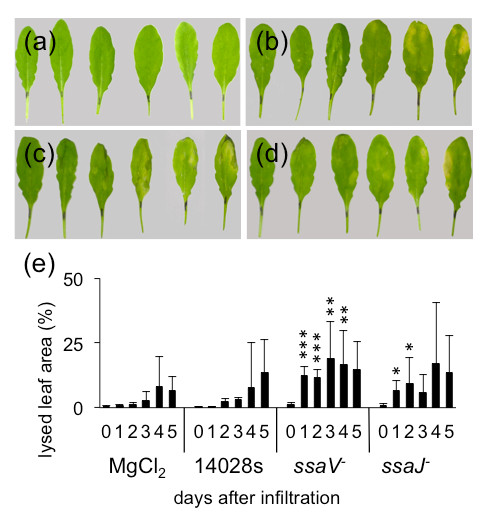
**Symptoms caused by T3SS-2 mutants.** Infection symptoms caused by the T3SS-2 mutants in *Arabidopsis* leaves. Wild type *Salmonella* or mutants in the SPI-2 encoded T3SS were infiltrated into Arabidopsis leaves; symptoms were analyzed during 5 DAI. a-d: Macroscopic observations of symptoms development from 0 to 5 DAI with **(a)**: 10 mM MgCl_2_ (buffer control), **(b)**: wild type 14028s strain, **(c)**: *ssaV*^−^mutant, **(d)**: *ssaJ*^−^mutant. **(e)**: Calculated average percentages of leaf surfaces showing infection symptoms. Five leaves per time point were photographed. Experiment was repeated five times. ∗*p* ≤ 0.05;∗∗*p* ≤ 0.005;∗∗∗*p* ≤ 0.0005 (Student’s *t* test).

**Figure 11:**
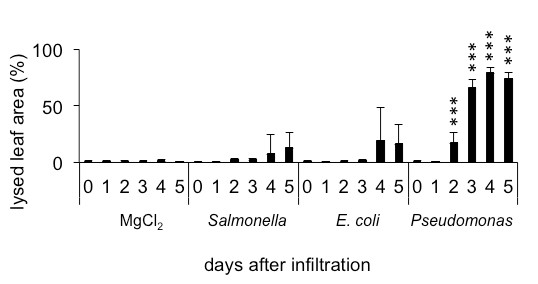
**Symptoms caused by virulent and avirulent bacteria.** Comparison of symptoms caused by the virulent *Salmonella* wild type 14028s, the non-pathogenic *E. coli* K12 and the plant pathogen *Pseudomonas syringae*. Average symptoms were calculated on the base of photos taken during 5 DAI. Five leaves per time point were analyzed, experiments were repeated 5 times. ∗*p* ≤ 0.05;∗∗*p* ≤ 0.005;∗∗∗*p* ≤ 0.0005 (Student’s *t* test).

Our recent results suggest that T3SSs play a significant role in virulence towards *Arabidopsis*[43]. We showed that mutants compromised in both *Salmonella* T3SSs proliferate slower in *Arabidopsis* leaves than the 14028s wild type bacteria [43]. *Salmonella* makes use of SPI-1 and SPI-2 T3SSs injecting several effectors with different functions at different stages of the infection [55,56]. Here, we wondered whether the reduced virulence is reflected in symptoms caused by those mutants in *Arabidopsis* plants and whether those symptoms can be used for automatic screening/analysis purposes. To this end, two mutants in SPI-1 encoded T3SS (*prgH*^−^and*invA*^−^) and two mutants in SPI-2 encoded T3SS (*ssaV*^−^and *ssaJ*^−^) were infiltrated into *Arabidopsis* leaves. Subsequently lesions were evaluated during 5 following days and expressed as percentage of total leaf surface. Infiltration with SPI-1 T3SS mutants (*prgH*^−^ and *invA*^−^) showed stronger symptoms from the first day onwards, if compared to infection with the wild type 14028s *Salmonella * (Figure 9). PrgH and InvA proteins are the parts of the outer and inner membrane-spanning rings of the *Salmonella* T3SS-1 apparatus respectively [55,57-59]. Similarly, SPI-2 T3SS mutants (*ssaV*^−^ and *ssaJ*^−^) provoked also stronger symptoms on *Arabidopsis* leaves than the 14028s wild type strain (Figure 10). SsaV and SsaJ proteins are necessary for constructing the core T3SS apparatus inside and outside of the bacterial membranes [55]. The infiltration experiments suggest the ability of wild type *Salmonella* Typhimurium to suppress the plant immune system by lowering the manifestation of hypersensitivity response (HR) to a level observed after infiltration with *E. coli* (Figure 11). A comparison between infection with non-pathogenic *E. coli* DH5*α* and highly pathogenic *Pseudomonas syringae* DC3000 showed significant lesions in *Pseudomonas*-infiltrated leaves and relatively mild symptoms in *E. coli*-infiltrated leaves (Figure 11). *Pseudomonas syringae* infiltrated *Arabidopsis* leaves showed necrosis and dark color patches.

### T3SS mutants cannot suppress the induction of the pathogenesis-related gene *PDF1.2*

**Figure 12:**
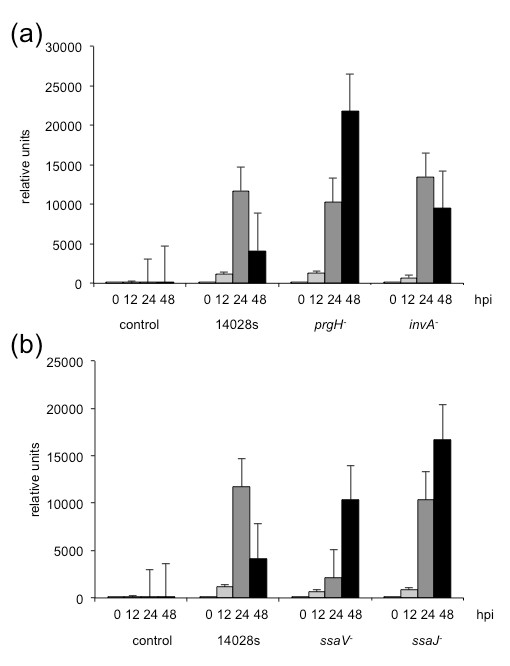
**Expression pattern of *PDF1.2*.** Expression pattern of *PDF1.2* gene in *ArabidopsisCol*-0 plants challenged with wild type *Salmonella* or T3SS mutants. Total RNA was extracted from 2-week-old seedlings inoculated with bacteria for hours as indicated. Relative expression levels of *PDF1.2* were normalized to the expression of *UBQ* gene. **(a)**: Transcriptional response to the T3SS-1 mutants *prgH*^−^and*invA*^−^. **(b)**: Transcriptional response to the T3SS-2 mutants *ssaV*^−^and*ssaJ*^−^.

In order to verify the observed suppression of plant immune responses we analyzed the expression level of the *PDF1.2* gene, which is known to respond to *Salmonella* challenge [20]. Fourteen-day-old *Arabidopsis* plants, grown on MS/2 agar medium, were transferred to liquid MS/2 medium 24 hours before bacterial inoculation. Wild type *S*. Typhimurium 14028s and *prgH*^−^, *invA*^−^, *ssaV*^−^ and *ssaJ*^−^mutants were grown on liquid LB medium with respective antibiotics, centrifuged and washed in 10 mM MgCl_2_. MS/2 medium containing the plants was inoculated with bacteria with final OD_600_ = 0.1. Whole plant materials were collected at 0, 12, 24 and 48 hours post inoculation. Quantitative reverse transcription PCR (qPCR) was done with *PDF1.2* primers and normalized to the expression of the *UBQ4(At5g25760)* housekeeping gene. Figure 11a-b shows the relative expression of *PDF1.2* gene after challenge with T3SS-1 (Figure 12a) and T3SS-2 (Figure 12b) mutants in comparison to the challenge with the 14028s wild type bacteria. The wild type *S.* Typhimurium strain 14028s showed its potential to decrease the expression of *PDF1.2* in *Arabidopsis* after the initial 24 hours induction. However, all of the mutants used in the study, except *invA*^−^, showed their inability to inhibit the plant defense, which is indicated by the increased expression of *PDF1.2* in *Arabidopsis*. These results are in line with the hypothesis that *Salmonella* suppresses the plant defense systems using T3SSs.

### Infection with T3SS mutant results in longer activation of MAP kinases

**Figure 13:**
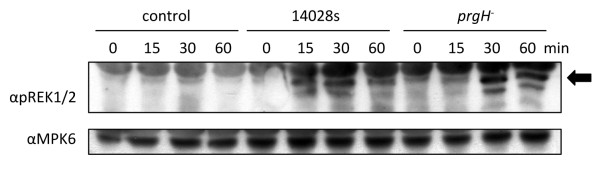
**Activation of MAPKs.** Phosphorylation status of MPK3 and MPK6 after treatment with *Salmonella*. Two-week-old seedlings were treated with bacteria for minutes as indicated. Total proteins were extracted and separated on SDS-PAGE. Phosphorylated form of MAK3/6 were detected using the anti pERK1/2 antibody (*α*pERK1/2) upper gel, the loading was done using *α*MPK6 antibody on parallel membrane loaded with equal amount of proteins (20 *μ*g). Arrows indicates the 43kDa band representing the phosphorylated form of MPK6.

MAP kinases are activated in plants by numerous pathogens, including *Salmonella*[20,60]. Activation of MAP kinases 3 (MPK3) and MPK6 pathways restricts *Salmonella* proliferation in *Arabidopsis*[20]. In order to demonstrate the activation of AtMPK3 and AtMPK6, the phosphorylation status was tested with an antibody against the phosphorylated form of the mammalian homologue: the extracellular-signal regulated kinases (ERK) 1/2. An inoculation experiment with 14028 s wild type and SPI-1 *prgH*^−^ mutant was performed and activation checked at different time points after inoculation (Figure 13). *S.* Typhimurium 14028 s as well as the SPI-1 mutant were found to activate the MAP kinases at 15 and 30 minutes after infection (MAI) (43kDa and 42kDa bands), the signal decreases however at 60 MAI. After infection with the SPI-1 mutant the initial activation at 15 and 30 MAI, remained until 60 MAI. This suggests the necessity of T3SS in the suppression of the plant MAP kinase signaling by *Salmonella*.

## Discussion

Plants have sophisticated mechanisms by which they recognize pathogen-originated signals. In case of pathogen attack, plants might initiate a rapid and intense activation of defense reactions known as hypersensitive response (HR). HR occurs within few hours and results in localized cell death. Very often HR is the consequence of effector-triggered immunity (ETI), which occurs when the plant recognizes the effectors injected by the pathogen into the plant cells. Rapid cell death or HR prevents the bacteria from spreading systematically. *Salmonella* uses diverse effectors to manipulate the cellular signals leading to the host defense response [42]. *Salmonellaenterica* subsp. *enterica* used in this study possesses two different T3SS, encoded by Salmonella Pathogenicity Island 1 (SPI-1) and SPI-2. Both T3SSs secret different yet overlapping sets of effector proteins tat function at different stages of the infection. However, many of the secreted effectors can by translocated via both T3SSs. The stronger symptoms seen in the leaves treated with the T3SSs mutants if compared to the wild type *Salmonella*, indicates the inability of *Salmonella* mutants to inhibit the molecular mechanisms that finally lead to HR, and in consequence it suggests the necessity of such effectors (and both functional T3SSs) for the infection of vegetal hosts. It is probable that both T3SSs are needed for the immune suppression, however the effectors translocated by the remaining T3SS in a mutant are sufficient to elicit ETI. Giving the importance for human health, the suppression of the animal immune system by *Salmonella* is very intensely studied. We know already 44 effectors which are injected by *Salmonella* into animal host cells, and for many of them we know the function and the target proteins [42]. Interestingly, very often bacterial effectors target the MAPK cascades, which are important regulators of the immune response in animals and plants. SpvC from *Salmonellaspp.* encodes a phosphothreonine lyase that dephosphorylates the pTXpY double phosphorylated activation loop in the ERK1/2 kinases [61-63]. Another effector from *Salmonellaspp.* the SptP inhibits phosphorylation and membrane localization of Raf kinase and therefore the activation of the downstream ERK kinases [64]. Although several *Salmonella* effectors have homologues in plant pathogenic bacteria, the SpvC is present in the *Pseudomonas spp.* as HopAI1, HopAO1 also from *Pseudomonas spp.* on the other hand, is the homologue of SptP, the function of *Salmonella* proteins in the inactivation of the plant immune system remains unknown. It is however very tempting to speculate that biochemical features of those effectors are conserved between animal and plant hosts, providing *Salmonella* (and other pathogenic bacteria) with efficient tools for suppression of the host immune system. Such suppression was reported in two recent reports. Shirron and Yaron studied infection of tobacco plants with *S.* Typhimurium [34]. The authors showed that in contrast to wild type living bacteria, dead bacteria elicited an oxidative burst and pH changes in tobacco cells. Similar response was provoked by the *invA*^−^ mutant, which has no functional SPI-1 T3SS [34]. Those results suggest that *Salmonella* depends on the secretion of effectors during infection of tobacco leaves to actively suppress their immune responses. A general transcriptome analysis performed in our laboratory suggests a similar scenario [43]. Infection with the *prgH*^−^ mutant, but not with the 14028 s wild type, induces about 640 genes, the majority of which are related to defense responses. Moreover, we showed that mutants impaired in their T3SSs are less virulent towards *Arabidopsis* plants then wild type bacteria [43]. Taken together, recently published and presented results build a growing body of evidences indicating that *Salmonella*, similarly to the infection in animals, actively suppresses the plant defense mechanisms. Whether this bacterium uses the same or different effectors in order to achieve this goal is not yet clear, it seems however to be acceptable to conclude that *Salmonella* uses the same T3SSs in plant and animal infections.

## Conclusions

This report presents an automatic pixel-based classification method for detecting “unhealthy” regions in leaf images. This method has been tested extensively with very promising results. Linear SVM has been used to classify each pixel. We have also shown how the results from SVM could be remarkably improved by using the neighborhood-check technique. The proposed method was compared to existing method and showed a higher accuracy. We used this algorithm to study the impact of the human pathogenic bacterium *Salmonella* Typhimurium on plants immune system. The comparison between wild type bacteria and T3SS mutants showed similarity in the infection process in animals and in plants. The result obtained with the proposed algorithm and also transcriptome and biochemical analyses suggest that T3SSs are necessary for a successful infection of plants. Plant epidemiology is only one possible application of the proposed algorithm, it can be easily extended to other detection tasks, which also rely on color information, or even extended to other features.

## Methods

### Plant growth

*Arabidopsisthaliana* wild type *Col*-0 (NASC ID: N70000) seeds were germinated on $\frac{1}{2}$ MS media for around 2 weeks. The seedlings were then transferred to soil and grown in short day chamber (7 hours of light) at 24°C for additional 4 weeks.

### Bacterial growth

*Salmonella enterica* subsp. *enterica* serovar Typhimurium (ATCC 14028s), *Salmonella* T3SS mutants (all in the 14028s genetic background) and *Escherichia coli* K12 strain DH5*α*were grown on LB agar and liquid media with required antibiotics. *Pseudomonas syringae* pathovar *tomato* DC3000 was grown in King’s B medium containing required antibiotics. *prgH*^−^ and *ssaV*^−^mutants were obtained from Prof. David Holden, Imperial College, London. *invA*^−^ and *ssaJ*^−^ mutants were constructed in the INRA Tours laboratory by Dr. Isabelle Virlogeux-Payant.

### Leaf infiltration

Around 6-week-old *Arabidopsis* plants were chosen for infiltration experiment. The cultured bacteria were spun down, washed with 10 mM MgCl_2_ solution. Final optical density (OD_600_) of infiltration solution was 0.1. Infiltration was done *via* syringe on the abaxial surface of the leaves.

### Analysis of lesions in leaves

For the analysis, images of leaves were captured at 5 consecutive days after infiltration. At least 5 leaves were photographed per each time point and infiltration variant. This experiment was repeated 5 times. Lesions in leaves were analyzed with the help of an automated program calculating the changed color in a proportion to the normal color of the leaves. The diseased portion were calculated in percentage and evaluated, cf. Section Image-Based Classification. Altogether over 1200 images were evaluated.

### Bacteria inoculation

Around 2-week-old *Arabidopsis* plants were transferred to $\frac{1}{2}$ MS liquid media and left undisturbed overnight. Bacteria were washed in 10 mM MgCl_2_, and the liquid medium was inoculated with bacteria at OD_600_ = 0.1. Whole plants were collected at regular intervals for further analysis.

### RNA extraction and reverse transcription

Extraction of total RNA was done with Trizol^*Ⓡ*^(Invitrogen) accordingly to manufacturer instructions. Whole plants were collected in liquid nitrogen and homogenized. Total RNA was extracted. All RNA samples were treated with DNase I (Fermentas International Inc.). Complementary DNA (cDNA) was prepared with the help of reverse transcriptase (qScript, Quanta Biosciences) accordingly to manufacturer protocol. Equal amount of 2 *μ*g RNA from all samples was taken to ensure the best possible gene expression levels analysis.

### Quantitative PCR

After the preparation of cDNA, quantitative PCR was performed in the Applied Biosystems 7500 FAST real-time PCR system. SYBR green was used as a fluorescence dye for the PCR reactions. 20 *μ*l total volume reaction was used and three repetitions were made for each of the sample. qPCR was done with the following primers: *UBQ*4: forward primer: GCT TGG AGT CCT GCT TGG ACG, reverse primer: CGC AGT TAA GAG GAC TGT CCG GC; *PDF1.2*: forward primer: GTT TGC TTC CAT CAT CAC CC, reverse primer: GGG ACG TAA CAG ATA CAC TTG.

### Western blot analysis

Whole plants were collected in liquid nitrogen, homogenized in a tissue homogenizer and total protein were extracted in 200 *μ*l of lysis buffer (25 mM TRIS (pH = 7.8), 10 mM MgCl_2_, 15 mM EGTA, 75 mM NaCl, 1 mM DTT, 0.5 mM NaVO_4_, 1 mM NaF, 15 mM *β*-glycerophosphate (Sigma-Aldrich), 15 mM 4-nitrophenyl phosphate (Sigma), 0.5 mM PMSF, 5 *μ*g/ml leupeptine (Roche), 5 *μ*g/ml aprotinin (Roche), 0.1% Tween 20). After vigorous vortexing, samples were centrifuged at 14,000 rpm and supernatant, containing the proteins was collected. Bio-Rad mini format 1-D electrophoresis system was used for sodium dodecyl sulphate polyacrylamide gel electrophoresis (SDS-PAGE). 12% resolving gel and 3.2% stacking gel were used. Equal amount of proteins (20 *μ*g) was used for each sample. Primary antibodies: *α*-phospho-ERK 1/2 (Sigma-Aldrich), AtMPK6 (Biolabs). Secondary antibody: Anti-Rabbit IgG HRP-conjgate (Sigma-Aldrich).

## Competing interests

The authors declare that they have no competing interests.

## Authors’ contributions

MS developed the support vector machine (SVM) and done the programing steps. BN and SM performed the experiments and done the calculations. MS, WK, DC, HH, KHK and AS conceived and discussed the project. MS, HH, KHK and AS wrote the publication. All authors have read and approved the final manuscript.

## Supplementary Material

Additional file 1 **Figure S1.** Training data. Scatter plot of the used training data. Only the color channels I2 and I3 are depicted. The healthy points are marked as green squares. The blue circles correspond to unhealthy training pixels. The background pixels are visualized with red crosses. Click here for file
