# Supplementary figures and images for: An image classification approach to analyze the suppression of plant immunity by the human pathogen Salmonella Typhimurium
Source: BMC Bioinformatics. 2012 Jul 19;13:171. doi: 10.1186/1471-2105-13-171 (PMC3519609; doi:10.1186/1471-2105-13-171)

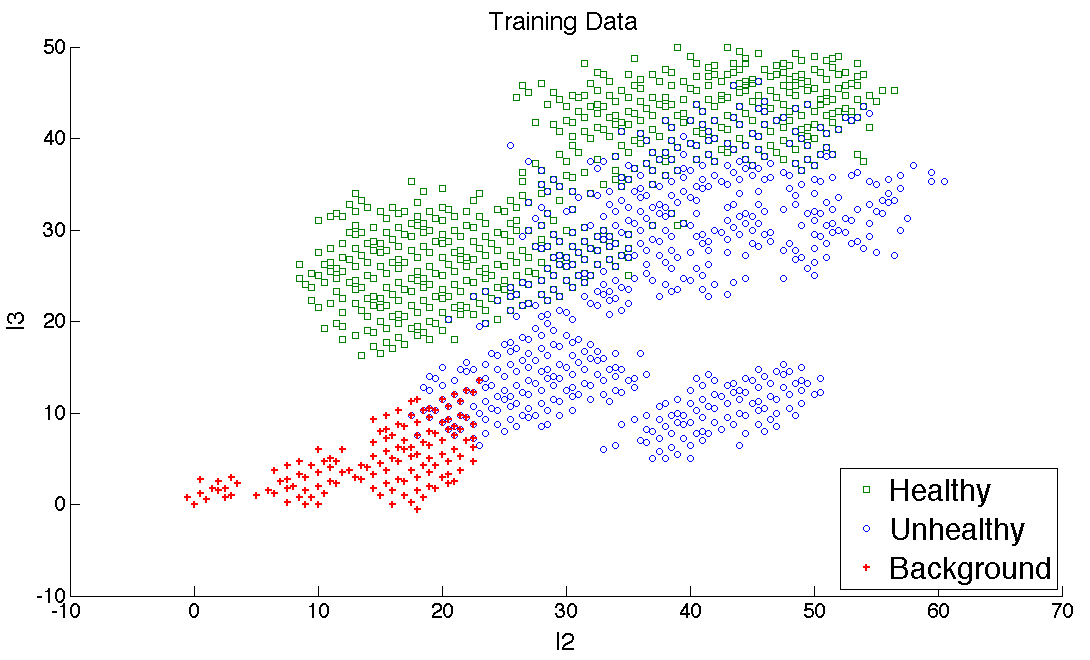

Supplement: Additional file 1 — Figure S1. Training data. Scatter plot of the used training data. Only the color channels I2 and I3 are depicted. The healthy points are marked as green squares. The blue circles correspond to unhealthy training pixels. The background pixels are visualized with red crosses. [file 1471-2105-13-171-S1.png]
